# Metformin in adults with type 1 diabetes: Design and methods of REducing with MetfOrmin Vascular Adverse Lesions (REMOVAL): An international multicentre trial

**DOI:** 10.1111/dom.12840

**Published:** 2017-02-17

**Authors:** John R. Petrie, Nish Chaturvedi, Ian Ford, Irene Hramiak, Alun D. Hughes, Alicia J. Jenkins, Barbara E. Klein, Ron Klein, Teik Chye Ooi, Peter Rossing, Naveed Sattar, Coen D. A. Stehouwer, Helen M. Colhoun, on behalf of the REMOVAL Trial Team, I Ford, S Kean, E Thomson, L Gillespie, J Gibb, N Greenlaw, I Hramiak, A Keech, A Jenkins, N Chaturvedi, A Hughes, K March, E Coady, T Tillin, M Bots, R Klein, B Klein, J Dreyer, T Jan, S Meuer, D Murach, Koby Sheffy, Ravit Lusky, S Peleg, J Petrie, H Colhoun, A Shore, D Carty, P Donnan, M Witham, A Adler, E Lonn, P Rauchhaus, I Hramiak, R Lindsay, M Brouwers, J Van‐Melckebeke, L Gillespie, T Hamill, L Cuthbertson, A Murray, L Jolly, E Miller, N Sattar, J Hair, A Bell, S Carmichael, E Douglas, P Surtees, E Dinnett, J Allan, S Kean, C Watson, M McLaughlin, G Brindley, E Smillie, D Motherwell, S MacDonald, P Ellis, D Stuart, M Travers, S Brearley, L Greig, J Haw

**Affiliations:** ^1^ Institute of Cardiovascular and Medical SciencesUniversity of GlasgowGlasgowUK; ^2^ Institute of Cardiovascular ScienceUniversity College LondonLondonUK; ^3^ St Joseph's Health CareLondonOntarioCanada; ^4^ NHMRC Clinical Trials CentreUniversity of SydneyAustralia; ^5^ Department of Ophthalmology and Visual Sciences, University of Wisconsin School of Medicine and Public HealthMadisonWisconsin; ^6^ Ottawa Hospital Research InstituteThe Ottawa HospitalOttawaCanada; ^7^ Steno Diabetes Center and the University of CopenhagenCopenhagenDenmark; ^8^ Department of Internal Medicine and Cardiovascular Research Institute Maastricht (CARIM)Maastricht University Medical CentreMaastrichtThe Netherlands; ^9^ Institute of Genetics and Molecular MedicineUniversity of EdinburghUK

**Keywords:** adjunct therapy, cardiovascular, carotid intima media thickness, clinical trial, complications, endothelial function, hypoglycaemia metformin, type 1 diabetes, weight

## Abstract

**Aims:**

Cardiovascular (CV) disease is a major cause of reduced life expectancy in type 1 diabetes (T1D). Intensive insulin therapy prevents CV complications but is constrained by hypoglycaemia and weight gain. Adjunct metformin reduces insulin dose requirement and stabilizes weight but there are no data on its cardiovascular effects. We have therefore initiated an international double‐blind, randomized, placebo‐controlled trial (REMOVAL: REducing with MetfOrmin Vascular Adverse Lesions in type 1 diabetes) to examine whether metformin reduces progression of atherosclerosis in adults with T1D. Individuals ≥40 years of age with T1D for ≥5 years are eligible if they have ≥3 of 10 specified CV risk factors. The enrolment target is 500 participants in 17 international centres.

**Materials and methods:**

After 12 weeks of single‐blind placebo‐controlled run‐in, participants with ≥ 70% adherence are randomized to metformin or matching placebo for 3 years with insulin titrated towards HbA1c 7.0% (53 mmol/mol). The primary endpoint is progression of averaged mean far wall common carotid intima‐media thickness (cIMT) measured by ultrasonography at baseline, 12, 24 and 36 months. This design provides 90% power to detect a mean difference of 0.0167 mm in cIMT progression between treatment arms (α = 0.05), assuming that up to 20% withdraw or discontinue treatment. Other endpoints include HbA1c, weight, LDL cholesterol, insulin requirement, progression of retinopathy, endothelial function and frequency of hypoglycaemia.

**Conclusion:**

REMOVAL is the largest clinical trial of adjunct metformin therapy in T1D to date and will provide clinically meaningful information on its potential to impact CV disease and other complications.

## 1 INTRODUCTION

Period life expectancy in people with type 1 diabetes (T1D) is reduced by 11 to 13 years^1^; rates of CV events are at least double those in the general population and account for approximately 45% of deaths.^2^ Long‐term post‐randomization data from the Diabetes Control and Complications Trial (DCCT) participants followed up in the Epidemiology of Diabetes Interventions and Complications (EDIC) study demonstrate that intensive blood glucose control reduces both microvascular and CV complications in T1D.^3^

However, in population‐based data only approximately 30% of individuals with T1D are near target HbA1c (<7.5%/59 mmol/mol) and at least 30% have poor control (HbA1c ≥ 9.0%/75 mmol/mol).^4^ A key barrier to optimizing glycaemia is hypoglycaemia; in the DCCT, rates of severe hypoglycaemia were 3‐fold higher in those randomized to intensive therapy and with HbA1c at or below target.^5^ Another long‐term issue is insulin‐induced weight gain, which may be accompanied by escalating insulin dose requirements, increased LDL cholesterol and/or raised blood pressure (BP).^6^, ^7^, ^8^

Adjunct therapy with metformin reduces insulin dosage in T1D and may attenuate weight gain^9^, ^10^, ^11^, ^12^, ^13^; some clinicians already use it in this context. As metformin reduces CV disease in type 2 diabetes (T2D),^14^, ^15^, ^16^ and is recommended first‐line therapy in most international guidelines,^17^ we hypothesized that it might also provide CV protection in T1D.

In this largest and longest trial of metformin in T1D to date, we aim to gather data on cardiovascular and metabolic endpoints as well as key aspects of long‐term safety (eg, vitamin B12 status, lactic acidosis). Progression of carotid artery intima‐media thickness (cIMT) measured by ultrasonography is the primary endpoint as it was reduced by intensive glucose control in DCCT‐EDIC^18^; this was later validated by reduced CV events.^3^

## 2 MATERIALS AND METHODS

### 2.1 Objectives and endpoints

The primary objective of the REducing with MetfOrmin Vascular Adverse Lesions in type 1 diabetes (REMOVAL: clinicaltrials.gov NCT01483560) is to test in adults with T1D whether metformin added to insulin therapy (titrated towards target HbA1c 7.0%/53 mmol/mol) reduces progression of atherosclerosis in the common carotid artery (CCA), defined as within‐person change in bilateral averaged mean far wall carotid intima‐media thickness (cIMT) measured annually over 3 years.

For measurement of cIMT, the same ultrasound system and pre‐set image parameter settings (eg, depth, gain, persistence, dynamic range, post processing) are to be maintained at each site throughout the study. The reading centre (University College London [UK]) trains each site sonographer who then submits 5 accreditation scans. Right and left carotid arteries are interrogated in B mode with a 7.0 MHz or higher broadband linear array transducer with concurrent recording of a three lead ECG. A plaque screen (defined as focal thickening ≥1.5 mm or 50% greater than surrounding IMT) of the near and far walls of the CCA, bulb and internal carotid artery segments is performed also. Longitudinal images of the CCA are obtained at anterior, lateral and posterior angles using Meijer's arc during at least 5 cardiac cycles. Additional cIMT measurements are performed on a panel of 6 participants at each site annually to monitor reproducibility. At the reading centre, triplicate measurements are taken from the distal centimetre of the CCA (ie, immediately proximal to the bulb) by a single trained assessor using a validated semi‐automated program.^19^ The assessor undergoes repeated “masked” QC cycles to assess repeatability.

For the assessment of retinal disease, 2 colour 45° field photographs (field 1, optic disc; field 2, macula) are taken in each eye at randomization and at 36 months. In the UK these are acquired directly from national retinal screening systems. Images are graded using custom‐designed software at the University of Wisconsin Ocular Epidemiology Reading Center (OERC) using the modified Airlie House classification scheme and the Early Treatment Diabetic Retinopathy Study severity scale as previously described.^20^ Component retinal lesions are evaluated individually. If significant retinal pathology exists, the site principal investigator is notified to ensure appropriate clinical action.

Endothelial function is assessed in centres covering 80% of participants using peripheral Arterial Tonometry (ENDOPAT, Itamar, Israel) to measure Reactive Hyperaemia Index (RHI) non‐invasively at 0, 12 and 36 months. This method assesses changes in digital pulse volume and pulse wave velocity.^21^ ENDOPAT studies are reviewed by Itamar staff and scan quality reported back to site staff within 1 week.

Other secondary and tertiary endpoints are shown in Table 1. Each of the secondary outcome measures will be analysed separately and the individual results will be reported. The protocol has a pre‐defined composite interpretation of the secondary outcomes where results will be considered clinically meaningful with the potential to influence clinical practice in the event that a statistically significant improvement in 2 or more of the following individual outcomes is observed on metformin: (1) HbA1c (by DCCT‐standardized local assays); (2) LDL‐cholesterol (centrally‐measured); (3) albuminuria, based on at least 2 separate urine specimens and routinely available assays (File S1, Appendix 3; (4) 2 or more step progression on the 11‐step modified concatenated retinopathy severity scale; (5) weight, by calibrated scales; (6) insulin dose; and (7) endothelial function (RHI).

**Table 1:** Study endpoints

**Change from baseline compared between treatment groups:**
**Primary**
Progression of averaged mean far wall common carotid artery IMT (CCA cIMT, measured in mm, at baseline, 12, 24 and 36 months).
**Secondary**
HbA1c (site DCCT‐aligned laboratories) LDL‐cholesterol (central lab) Albuminuria^1^ Retinopathy stage (2‐step progression on the ETDRS scale) Weight Insulin dose Endothelial function (in at least 80% of participants)
**Composite interpretation of all secondary endpoints**
Improvement in 2 or more of these secondary endpoints will be considered clinically meaningful with the potential to influence clinical practice.
**Tertiary**
Frequency of hypoglycaemia *(modified Steno Hypoglycaemia Questionnaire)* Treatment satisfaction *(Diabetes Treatment Satisfaction Questionnaire)* Markers of endothelial function (t‐PA, sE‐selectin, sICAM‐1) Progression of averaged maximal distal common carotid artery IMT (CCA cIMT, measured in mm, at baseline, 12, 24 and 36 months). Vitamin B12 status

^1^ Time to event analysis using a Cox Proportional Hazards Model.

### 2.2 Trial management

The protocol was approved by the West of Scotland Research Ethics Service (REC1) (UK) and the following Medical Research Ethics Committees/Institutional Review Boards: St Vincent's Hospital and Royal Melbourne Hospital (Melbourne) and Royal Prince Alfred Hospital (Sydney, Australia; Western University Health Science Research Ethics Board (Canada); Hovedstaden Region Centre of Health (Denmark); and Maastricht University Medical Centre, (Netherlands). Trial governance and oversight is the responsibility of the co‐sponsors (University of Glasgow and Greater Glasgow and Clyde Health Board, UK) with trial monitoring outside the UK and Denmark delegated by agreement to national partner institutions. Active and matching placebo study medications are provided free of charge by Merck KGaA (Darmstadt, Germany). All scans and photographs are uploaded by site personnel via a purpose‐designed electronic Case Report Form (eCRF) on to a secure server at the University of Glasgow for digital archiving and subsequent download for analysis at reading centres. Data management is by the Robertson Centre for Biostatistics, University of Glasgow. An Independent Data Monitoring Committee (IDMC) reviews 6‐monthly unmasked reports on study progress. Seventeen initial and 5 reserve sites with expertise in cIMT measurement have been selected across the UK, Australia, Canada, Denmark and the Netherlands. Where long‐term post‐randomization follow‐up is permitted, we seek consent from participants for the local team to remain in contact at trial end.

### 2.3 Screening, eligibility, enrolment and run‐in period

Individuals aged ≥40 with ≥5 years T1D and at least 3 of 10 specified CV risk factors are eligible (Table 2). T1D is defined as diagnosis of diabetes before age 35 years and insulin use within 1 year. Potential participants are approached by mail or personally at regular clinic visits; those expressing an interest are given further information and invited to return to a (non‐fasting) screening visit.

**Table 2:** Entry criteria^1^

Inclusion	Exclusion
Type 1 diabetes for 5 years or more^2^	eGFR < 45 mL/min/1.73 m^2^ Woman of childbearing age not using effective contraception Pregnancy and/or lactation Acute Coronary Syndrome or Stroke/TIA within the last 3 months NYHA stage 3 or 4 heart failure Uncontrolled angina Significant hypoglycaemia unawareness^6^ Impaired cognitive function/unable to give informed consent Previous carotid surgery/inability to capture adequate carotid images Gastroparesis^6^ History of lactic acidosis Other contraindications to metformin Hepatic impairment Known hypersensitivity to metformin Acute illness (dehydration, severe infection, Shock, acute cardiac failure) Suspected tissue hypoxia Any co‐existent life threatening condition including prior diagnosis of cancer within 2 years History of alcohol problem or drug abuse
Age ≥ 40 years	
7.0 ≤ HbA1c < 10.0% (53‐86 mmol/mol)	
AND	
*3 or more* of the following 10 CVD risk factors	
BMI ≥ 27 kg/m^2^ Current HbA1c > 8.0% (64 mmol/mol) Known CVD/peripheral vascular disease Current smoker eGFR < 90 mL/min/1.73 m^2^ Confirmed micro‐ (or macro‐) albuminuria^3^ Hypertension (BP ≥ 140/90 mm Hg; or established antihypertensive treatment) Dyslipidaemia^4^ Strong family history of CVD^5^ Duration of diabetes >20 years	

^1^ Abbreviated from full Protocol Version 1.0.

^2^ Defined as diagnosis below age 35 years AND insulin use within 1 year of diagnosis.

^3^ As judged by the site principal investigator based on at least 2 urine samples assayed locally and interpreted according to site reference ranges (File S1, Appendix 3).

^4^ Total cholesterol ≥5.0 mmol/L (200 mg/dL); or HDL cholesterol <1.2 mmol/L (46 mg/dL) [men] or <1.3 mmol/L (50 mg/dL) [women]; or triglycerides ≥1.7 mmol/L (150 mg/dL); or established on lipid‐lowering treatment.

^5^ At least one parent, biological aunt/uncle, or sibling with myocardial infarction, or stroke aged <60 years).

^6^ Confirmed as significant by site principal investigator.

Following informed consent, past medical history, family history and concomitant medication (including duration, type and dose of any previous statin and/or ACE inhibitor therapy) are recorded on the eCRF. Height, body weight, ethnicity and smoking status are documented; blood pressure (BP) and heart rate are measured in triplicate according to Standard Operating Procedures specified in the protocol. The Steno Hypoglycaemia questionnaire (File S1) and the Diabetes Treatment Satisfaction Questionnaire (DTSQ)^22^ are administered. Blood and urine samples are sent to local laboratories for measurement of HbA1c, serum lipids, liver function tests, albuminuria, renal function (unless results are available from the previous 90 days) and random C‐peptide. Aliquots of serum, plasma, urine and buffy coat are retained for biomarker assays and later DNA extraction. Cholesterol and BP lowering therapies are reviewed against local standard of care and treatment adjusted as indicated. Urine for pregnancy testing is requested from women of childbearing potential who are not using an effective method of contraception at this (and all subsequent) in‐person visits, with a view to discontinuing study medication if the test is positive.

**Figure 1:**
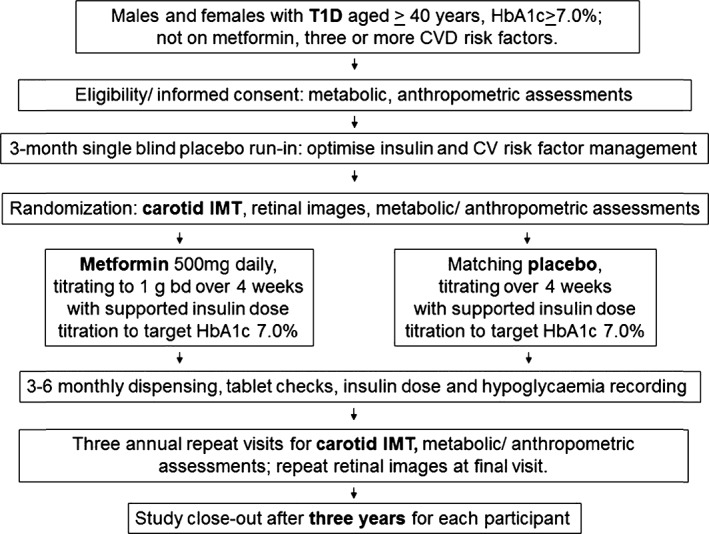
Outline of protocol.

Enrolled participants are invited to enter a 3‐month run‐in period and receive single‐blind placebo tablets (matching metformin 500 mg) to take once daily with the evening meal during the third month only (Figure 1). The first of a series of dedicated study diaries is provided, containing guidance on study medication dose titration, adverse effects and “sick day rules” as well as contact details for the local study team. Space is provided for structured recording of insulin doses and 4‐point blood glucose profiles during 3 days prior to each scheduled telephone or in‐person visit. Participants are also asked to record all changes in concomitant medication.

Individual insulin regimens are reviewed by site staff at the beginning of the run‐in period with a view to making any changes required to facilitate optimisation of control (target HbA1c 7.0%/53 mmol/mol). Additional in‐person clinical visits are arranged if necessary. Structured telephone visits are conducted approximately monthly during the remainder of the run‐in, for review of blood glucose monitoring data and insulin doses; this support remains available throughout the trial.

### 2.4 Randomization and follow‐up

A randomisation visit (fasting) is scheduled at the end of the run‐in period. Participants with ≥70% adherence and a screening visit C‐peptide ≤0.2 nmol/L undergo baseline study measurements including cIMT, endothelial function and retinal photographs. Randomization is by an Interactive Voice Response System (IVRS) hosted by the Robertson Centre for Biostatistics. Double‐blind study medication (metformin as Glucophage 500 mg or matching placebo) is issued in identical packages covering 3‐ or 6‐month periods according to the visit schedule.

Participants are asked to carry a Patient Alert Card containing details of emergency unmasking procedures. Following randomization, they are asked to up‐titrate their dose of study medication on a weekly basis from 1 tablet daily with the evening meal in week 1, to 2 tablets per day (with breakfast and evening meal) in week 2, until the target dose of 2 tablets with each of these meals (equivalent to 1000 mg twice daily) is achieved in week 4. Dose titration is supported by weekly telephone visits; dose down‐titration or treatment interruption (eg, in response to gastrointestinal adverse effects) is permitted at any time during the trial. If treatment interruption persists for more than 4 weeks, site staff are instructed to record a permanent treatment discontinuation. Treatment restart is encouraged at any time, if appropriate, at the discretion of the site principal investigator. Current dose is recorded at each in‐person visit with tablet counts conducted by site staff.

As far as possible, study visits are designed to coincide with appointments in routine care; repeat assessments of the main study endpoints and other items are conducted at 12, 24 and 36 months (Table 3). During the trial, all participants continue to have access to the usual local arrangements for diet and lifestyle advice, along with weight management. Ongoing glycaemia, BP and cholesterol management are under the care of the site PI and the usual care team, according to updated national and international guidelines.

**Table 3:** Schedule of visits (abbreviated)

Activity		Run‐in period			Randomize	Months															
Month	Screen	‐ 12 weeks			0			1	3	6	9	12	15	18	21	24	27	30	33	36	
Telephone visit^1^	Visit	Visit	Visit	Visit	Visit	Visit	Visit	Visit	Visit	Visit	Visit	Visit	Visit	Visit	Visit	Visit	Visit	Visit	Visit	Visit	Closeout
^F^ Fasting visit	R1	R2^1^	R3^1^	R4^1^	1^F^	2^1^	3^1^	4^1^	5	6	7 ^1^	8 ^F^	9^1^	10	11^1^	12 ^F^	13^1^	14	15^1^	16 ^F^	
Informed consent	x																				
Randomization					x																
Current medications	x				x			x	x	x	x	x		x		x		x		x	
Height, weight	x				x				x	x		x		x		x		x		x	
Assess insulin dose		x	x	x	x	x	x	x	x	x	x	x	x	x	x	x	x	x	x	x	x
Questionnaires	x				x			x	x	x	x	x	x	x	x	x	x	x	x	x	
Blood Samples	x				x				x	x		x		x		x		x		x	
Pregnancy test	x				x	*Repeated if applicable*															
Carotid IMT					x							x				x				x	
Retinal images					x															x	
Endothelial function^1^ some centres only					x							x								x	
Urine sample	x				x							x				x				x	
Dispense study medication	x				x				x	x		x		x		x		x			

^1^ Telephone visit; ^*F*^fasting visit.

### 2.5 Hypoglycaemia

Participants are asked to record all symptomatic or biochemically‐proven hypoglycaemic episodes (<2.8 mmol/L; 50 mg/dL) in the study diary. This information is used by site nurses at follow‐up visits as a basis for completing the Steno Hypoglycaemia Questionnaire in which events are categorized as: minor (self‐treated, resolved with short acting glucose and longer acting carbohydrate); major (requiring assistance from 1 or more other persons); or major with unconsciousness (self‐reported).

### 2.6 Safety and pharmacovigilance

Hepatic and renal function is monitored at all in‐person visits. Permanent discontinuation of study medication is mandated in cases of significant hepatic impairment (alanine transaminase >3.0 times upper limit of normal) or renal impairment (eGFR < 30 mL/min/1.73 m^2^). Investigators are advised to reduce the study medication dose to 1 tablet twice daily in all participants in whom eGFR falls below 45 mL/min/1.73 m^2^ during follow‐up. Serum lactate is checked at baseline and annually; study medication is permanently discontinued if a single measurement is >5.0 mmol/L with acidosis (including routine clinical care) or if a level >3.0 mmol/L is sustained on a mandated repeat sample within 1 week. Vitamin B12 levels are monitored annually; participants in whom levels fall below 150 pmol/L are offered the choice of treatment discontinuation or referral back to primary care for injectable supplements.

In addition to serious adverse event reporting, specific gastrointestinal, neurological, metabolic, renal and cardiovascular adverse events of medical interest are also recorded, as well as new diabetes‐related complications, operations or procedures.

Following each meeting of the IDMC (see above), a recommendation is made to the co‐sponsors regarding the appropriateness of continuing the trial, from a safety and efficacy perspective. In addition to these arrangements, a Glycaemia Committee led by Dr Irene Hramiak (Ontario, Canada) sends detailed blinded reports of the participants’ HbA1c status and rates of hypoglycaemia to each site every 6 months, along with “benchmarking” data from other sites in their region. The Committee can contact and support centres in which average HbA1c is higher than that in other comparable centres.

### 2.7 Statistical considerations

All analyses will be conducted blinded to treatment allocation. The principal analysis will be on a modified intention‐to‐treat analysis set, ie, including all subjects from the intention‐to‐treat population (all randomized participants, regardless of subsequent participation in the study) with data available (without imputation). The target sample size is based on analysis of cIMT primary endpoint data using repeated measures regression analysis, assuming a linear progression in the control arm of mean 0.044 mm and SD 0.050 mm over 3 years.^23^ Regression model effect estimates with 95% confidence intervals and associated *P* values will be calculated. In order to minimize the residual SD, cIMT data will be adjusted for baseline cIMT as well as for age, sex and baseline levels of cardiovascular risk factors predictive of cIMT progression (specified in the Statistical Analysis Plan). To account for differences in ultrasound machines used at sites, a sensitivity analysis adjusting for ultrasound probe frequency is also specified along with a separate per protocol analysis.

A final sample size of 200 participants per treatment arm provides 90% power to detect an average mean cIMT difference of at least 0.0167 mm (one third of an SD) between treatment arms (α = 0.05); we therefore aim to recruit 500 patients allowing for 20% treatment withdrawal and/or treatment discontinuation. This sample size will provide 90% power to detect differences of approximately 0.3 SD in secondary endpoints including lipid, metabolic and endothelial function (α = 0.05). The retinopathy secondary endpoint is exploratory; if 3‐year 2‐step progression in the ETDRS category is estimated at 13.7%, treatment with metformin will necessarily be associated with a 60% reduction in risk for 80% power to declare significance at *P* < 0.05. No interim analyses are planned or pre‐specified.

## 3 RESULTS AND DISCUSSION

REMOVAL is the first adequately‐powered long‐term trial addressing the impact of metformin on a valid CV surrogate outcome (cIMT) in T1D. It will also collect data on metabolic endpoints (insulin dose, weight, HbA1c, hypoglycaemia, LDL cholesterol) as well as other vascular outcomes (endothelial function, retinal disease).

Metformin is a biguanide that undergoes active transport via cationic transporters and accumulates in intestinal cells.^24^ During steady‐state oral therapy, plasma glucose is reduced mainly by inhibition of hepatic glucose production.^25^ Glucose‐lowering is key in reducing microvascular complications in both T1D and T2D, but its effect on cardiovascular (macrovascular) complications is more complex, as other risk factors impact to a greater or lesser extent.

The differing molecular mechanisms of action of the various available classes of glucose‐lowering “antidiabetic” agents are critical to their overall therapeutic profile as candidates for adjunct therapy. For metformin, mechanisms relevant to glucose‐lowering include activation of AMP‐activated protein kinase (AMPK),^26^ inhibition of mitochondrial glycerophosphate dehydrogenase,^27^ and release of gut hormones (including glucagon‐like peptide‐1).^28^ However, other downstream effects of AMPK have been postulated to mediate vascular actions of metformin,^29^ including modulation of proinflammatory pathways in perivascular adipose tissue^30^ and inhibition of STAT3 (and thereby monocyte to macrophage differentiation) in vascular tissue.^31^ Moreover, metformin can inhibit advanced glycosylation end product (AGE) formation by binding and inactivating methyglyoxal via an AMPK‐independent pathway.^32^

The primary focus of REMOVAL is to assess metformin's effects on the cardiovascular system in adults with T1D who are at high risk of CV disease, rather than its ability to lower glucose levels. Accordingly, we adopted a double‐blind, placebo‐controlled, “treat to target HbA1c” design. When adjunct agents are prescribed in T1D, insulin doses are often down‐titrated to avoid hypoglycaemia, such that an overall effect on glycaemia (measured by HbA1c) is not sustained.^10^, ^11^, ^13^ Thus, although HbA1c is 1 of 7 pre‐specified secondary endpoints, it is unlikely, by design, that a sustained separation in glycaemia between active and placebo arms will be observed. Instead, the trial is powered to detect whether 3 years of treatment with metformin reduces atherosclerosis progression as measured by cIMT. In addition to measuring vascular structure, we are assessing endothelial function (RHI) in 80% of participants, to provide an index of vascular function.

cIMT can be considered a validated surrogate endpoint for atherosclerotic disease in T1D on the basis of DCCT‐EDIC.^3^, ^18^ However, despite the variety of pathways by which metformin has been hypothesized to exert potentially beneficial effects on the cardiovascular system, 29, 30, 31 there is conflicting evidence regarding its effects on cIMT. We recently reported that metformin had no impact on cIMT over 18 months in non‐diabetic patients with established coronary heart disease^33^; similarly, no reduction in cIMT progression was detected in insulin‐treated people with T2D in the recent (underpowered) Copenhagen IMT trial.^34^ However, metformin has been reported to reduce cIMT progression in metabolic syndrome^35^ and also in T2D.^36^ REMOVAL is the first cIMT progression trial concerning T1D; in this context it is important to note that mechanisms of accelerated atherosclerosis in T1D and T2D differ in a number of aspects.^37^, ^38^

Despite a paucity of evidence concerning T1D, metformin (embonate) already holds a product license for use in T1D in France^39^; moreover, the UK National Institute for Clinical Excellence (NICE) recently recommended metformin for adults with T1D and BMI ≥ 25 kg/m^2^ who “want to improve glucose control while minimising their effective insulin dose.”^40^ Currently, more than 50% of people with T1D are now obese or overweight.^8^ Given that REMOVAL is planned to be 3 times longer and larger than any previous T1D metformin trial, the secondary endpoint data will be of considerable clinical utility in addressing longer term metabolic effects (eg, those on weight and insulin dose).

As metformin is structurally related to phenformin, which was withdrawn in the 1970s because of cases of lactic acidosis, concerns have been expressed regarding its use in ketoacidosis‐prone T1D patients.^41^ Metformin is commonly associated with gastrointestinal adverse effects, and long‐term use in T2D is associated with vitamin B12 deficiency.^42^, ^43^ Rather than simply extrapolating its adverse effect profile and overall tolerability from T2D, REMOVAL will gather important specific safety data on metformin in T1D.

A key limitation of the study is use of a surrogate primary endpoint rather than clinical cardiovascular events.^44^ Although several large T2D CV outcome trials have been reported recently and many more are in progress,^45^ not a single randomized trial of any intervention with CVD as the primary outcome has been performed in T1D to date, despite the undoubted impact of CV disease in this condition.^1^, ^2^ Much of the current evidence base for CV preventive strategies in T1D (including that for statins) is extrapolated from T2D or from meta‐analysis of T1D subgroups.^46^, ^47^ Another limitation is that focusing on atherosclerosis progression in the carotid artery may obviate detection of any beneficial cardiovascular effects mediated by other mechanisms. Data on which to base estimates of the degree of cIMT disease and the rate of change in our population were limited (mainly from DCCT); thus, there remains a degree of uncertainty in the power of a 3‐year intervention study in this population. Finally, the retinal endpoint can be regarded only as exploratory; it was included to acquire a point estimate for any likely effect size to guide future research, given the relatively low marginal cost of acquiring images from routine screening (at least at UK sites).

Two different glucose‐lowering agents used in T2D have recently been demonstrated to improve cardiovascular outcomes: a GLP‐1 agonist^48^ and an SGLT2 inhibitor.^49^ We anticipate that our international effort in REMOVAL, the largest and longest clinical trial of adjunct metformin therapy concerning T1D to date, will illustrate the feasibility of conducting large collaborative multi‐centre cardiovascular trials of adjunct therapy in T1D. Whether the data for metformin in the REMOVAL trial are positive or negative, we hope they will provide a stimulus to funding agencies and the wider diabetes community to support timely trials of other adjunct therapy candidates, with the twin aims of improving metabolic control and CV outcomes. Agents that are able to reduce rates of CV disease are urgently needed in T1D.

### Conflict of interest

None of the authors report relevant competing interests.

### Author contributions

J. R. P. and H. C conceived the trial and are its Chief and Deputy Chief Investigators, respectively. J. R. P., N. C., I. F., A. H., A. J., B. K., R. K., P. R., N. S., C. D. A. S., and H. C. contributed to study design. J. R. P., N. C., I. F., A. H., A. J., B. K., R. K., T. C. O., P. R., N. S., C. D. A. S, and H. C. were involved in the conduct/ data collection of the trial. J. R. P. wrote the first draft of the manuscript; all authors provided input into revising and writing the final version.

## Supporting information


**Appendix S1.** Supplementary Appendix (Acknowledgements (study personnel), modified Steno Hypoglycaemia Questionnaire, and definitions of microalbuminuria).
