# Supplementary material for: Metformin in adults with type 1 diabetes: Design and methods of REducing with MetfOrmin Vascular Adverse Lesions (REMOVAL): An international multicentre trial
Source: Diabetes Obes Metab. 2017 Feb 17;19(4):509–16. doi: 10.1111/dom.12840 (PMC5357575; doi:10.1111/dom.12840)
Supplement: Supplementary file 1 — Appendix S1. Supplementary Appendix (Acknowledgements (study personnel), modified Steno Hypoglycaemia Questionnaire, and definitions of microalbuminuria). [file DOM-19-509-s001.docx]

**Supplementary Appendices**

**a) Appendix 1: Acknowledgements**

**Funding:** Juvenile Diabetes Research Foundation (New York, USA).

**Study medication:** Donated and shipped to sites by Merck Germany KGaA (Darmstadt, Germany).

**Steering Committee:** J.R. Petrie^+^ (Chair and Chief Investigator, University of Glasgow, UK); H. Colhoun (Deputy Chief Investigator: University of Dundee, UK); N. Chaturvedi (University College London, UK), Jonathan Haw (participant representative – now deceased), I. Ford (University of Glasgow, UK), I. Hramiak^+^ (University of Western Ontario, Canada), A. Hughes (University College London, UK), A. Jenkins^+^ (University of Sydney, Australia), B. Klein (University of Wisconsin, USA), R. Klein (University of Wisconsin, USA), T.C. Ooi (The Ottawa Hospital, Canada), P. Rossing^+^ (Steno Diabetes Center, Denmark), N. Sattar (University of Glasgow, UK), C. Stehouwer^+^ (University of Maastricht, Netherlands). H Nickerson (non-voting, JDRF representative) (^+^ = national Principal Investigator)

**Trial Coordination: Robertson Centre for Biostatistics, University of Glasgow, UK:** I Ford, S Kean, E Thomson, L Gillespie, J Gibb, N Greenlaw; **Robarts Research Institute** **(Ontario, Canada):** I Hramiak**; NHMRC Clinical Trials Centre, Sydney:** A Keech, A Jenkins; **Carotid Reading Centre (University College London, UK):** N Chaturvedi, A Hughes, K March, E Coady, T Tillin; **Carotid External Quality Assurance** **(Julius Centre for Health Sciences, Utrecht, Netherlands):** M Bots; Ocular Epidemiology Reading Center, **Department of Ophthalmology and Visual Sciences (University of Wisconsin, Madison, Wisconsin, USA):** R Klein, B Klein, J Dreyer, T Jan, S Meuer, D Murach (supported by an unrestricted grant from Research to Prevent Blindness; **ENDOPAT Centre (Itamar Medical, Israel):** Koby Sheffy, Ravit Lusky, S Peleg. **ENDOPAT Committee:** J Petrie (Glasgow), H Colhoun (Dundee), A Shore (Exeter), D Carty (Glasgow); **Data Monitoring Committee:** P Donnan (Dundee), M Witham (Dundee), A Adler (Cambridge), E Lonn (Toronto), P Rauchhaus *(DMC Statistician)*; **Glycaemia Committee:** I Hramiak (Ontario, CA), R Lindsay (Glasgow, UK), M Brouwers (Maastricht, NL); **Project Management Unit** (NHS Glasgow)**:** J Van-Melckebeke, L Gillespie, T Hamill, L Cuthbertson, A Murray, L Jolly, E Miller; **Biomarker Laboratory (University of Glasgow):** N Sattar. **Biorepository** (NHS Glasgow): J Hair, A Bell; **Drug Supply Management** (NHS Glasgow): S Carmichael, E Douglas, P Surtees; **Pharmacovigilance** (NHS Glasgow)**:** E Dinnett; J Allan, S Kean; **Data Monitoring (UK):** C Watson, M McLaughlin**,** G Brindley, E Smillie; **Financial Management:** D Motherwell, S MacDonald (Glasgow); **Contracts and Agreements:** P Ellis, D Stuart (University of Glasgow); M Travers (NHS Glasgow); **Scottish Diabetes Research Network:** S Brearley, L Greig. **Patient representative:** J Haw (London, UK) (deceased).

**Recruiting Centres and Site Staff: Australia:** Melbourne (Royal Melbourne Hospital), P Colman (PI), A Nankervis, S Fourlanos; D West; S Vaughan, M Bjorasen; *J Donlon*; Melbourne (St Vincent’s Hospital), D O’Neal (PI), J Horsburgh, H Pater, S Kent, *J Vrazas*; Sydney: (Royal Prince Alfred Hospital) S Twigg (PI), G Fulcher, A Keech, R Denner, *A Coy*. **Canada:** London, Ontario, I Hramiak (PI), S Tereschyn, N Schmidt, H Lyle, *M Weingert,* *S Burke; H Heard*; Ottawa, Ontario, TC Ooi (PI), H Lochnan (Co-PI), A Sorisky, E Keely, J Malcolm, J Maranger, C Favreau, *S Petherick, K Boles*. **Denmark:** Steno Diabetes Center, P Rossing (PI), TW Hansen, *B Hemmingsen*. **England:** Bristol (Bristol Royal Infirmary), N Thorogood (PI), K Green, *T Robinson*; Durham (University Hospital), K Abouglilia (PI), D Nayman, *C Miller*; Exeter, (Royal Devon and Exeter Hospital), R Warren (PI), *K Aizawa*; Gloucester (Gloucestershire Royal Hospital), Dr M Balasubramani (PI), S Toth, *K Harvey, G Birch*; Hull (Michael White Centre for Diabetes), T Sathyapalan (PI), A James, *Z Javed*; Liverpool (Aintree University Hospital), J Wilding (PI), B Martin, S Birch, P Rooney, *A Wilcox, N Watson*; London (St Mary’s Hospital), N Oliver (PI), N Jugnee, *K March*; Manchester (Central Manchester University Hospitals), M Rutter (PI), T Turgut (Co-PI), A Shaju, L James, S Yau, *S Subin*; Newcastle (Royal Victoria Hospital), M Walker (PI), D Wake, *C Miller*; Plymouth (Derriford Hospital), A Millward^+^ (PI), P Chong (PI), M Coles, *J George*. **The Netherlands:** Maastricht University Medical Centre, Professor Coen Stehouwer (PI), MC Brouwers (Co-PI), N Schaper, J Pinxt, *J op het Roodt.* **Scotland:** Aberdeen (Aberdeen Royal Infirmary), S Phillips (PI), L Murray, *Linda Sleigh*; Ayr (Ayr Hospital, A Collier (PI), J Cook, *K Campbell, L Hodge*; Dundee (Ninewells Hospital), G Leese, G Reekie, *K Shields*; Edinburgh (Royal Infirmary), A Jaap (PI), A Sudworth, *A White*; Edinburgh (Western General) J McKnight (PI), L Steven, *A White*; Glasgow (Stobhill Hospital), G McKay (PI), A Llano (deputy-PI), G Currie, E Lennon, J Johnstone, F Heggie, *K Shields. (Sonographers in italics)* (^+^ = national Principal Investigator).

**b) Appendix 2: Modified Steno Hypoglycemia Questionnaire**

**c) Appendix 3: Definitions of microalbuminuria**

For the presence or absence of microalbuminuria to be judged in relation to the inclusion criteria, the results of local assays conducted on at least two separate urine specimens must be available. The final decision will lie with the site Principal Investigator according to local protocols, guided by the following criteria.

| **Units** | **Definition** | |
| --- | --- | --- |
|  | **Male** | **Female** |
| **First morning sample** | | |
| mg/ mmol^1^ | ≥ 2.5 | ≥ 3.5 |
| mg/g^1^ | ≥ 25 | ≥ 35 |
| μg/ mg^1^ | ≥ 25 | ≥ 35 |
| mg/L* | ≥30 | |
|  |  |  |
| **Timed** | | |
| μg/ min | >20 | |
| mg/ 24 hours | >30 | |
|  |  |  |
| ^1^ACR = albumin: creatinine ratio;  ^*^simple concentration (not preferred method) | | |

Occurrence of new microalbuminuria during the trial will be judged according to local assays, the results of which will be recorded in the eCRF.

Central assays may later be performed on stored urine aliquots to support the microabuminuria secondary endpoint analysis.
